# The Action of Medicines on the System

**Published:** 1860-01

**Authors:** 


ART. VII.—*The Action of Medicines on the System.* By FREDERICK WILLIAM HEADLAND, M.D., B.A., F.L.S., Licentiate of the Royal College of Physicians, etc. etc. Third edition, revised and enlarged. 8vo., pp. 463. Lindsay & Blakiston: Philadelphia, 1859.

THIS is another of the excellent reprints of Messrs. Lindsay & Blakiston. The fact that the work, within a few years, has reached a third edition, is a sufficient evidence of the estimation in which it is held in Europe and this country. In apologizing for his delay in bringing out a new edition, the author states that it has been caused solely by his desire to spare no pains to render the essay as complete as possible. Some matters have been noticed which had not been before included, and several points of general interest have been discussed more fully than they had been previously. The new edition is issued in beautiful style, and reflects great credit upon the taste and enterprise of the American publishing house.

